# Rapid evolution of prehistoric dogs from wolves by natural and sexual selection emerges from an agent-based model

**DOI:** 10.1098/rspb.2024.2646

**Published:** 2025-02-12

**Authors:** David C. Elzinga, Ryan Kulwicki, Samuel Iselin, Lee Spence, Alex Capaldi

**Affiliations:** ^1^ Department of Mathematics & Statistics, University of Wisconsin-La Crosse, La Crosse, WI, USA; ^2^ Department of Mathematics, University of Tennessee, Knoxville, TN, USA; ^3^ Department of Mathematics & Statistics, Valparaiso University, Valparaiso, IN, USA; ^4^ School of Mathematics, Georgia Institute of Technology, Atlanta, GA, USA; ^5^ Department of Mathematics & Statistics, James Madison University, Harrisonburg, VA, USA

**Keywords:** proto-domestication hypothesis, agent-based model, wolf, dog, domestication, single-trait evolution

## Abstract

Wolves are among the earliest animals to be domesticated. However, the mechanism by which ancient wolves were domesticated into modern dogs is unknown. Most of the prevailing domestication hypotheses posit that humans selectively bred wolves that were more docile. However, a competing hypothesis states that wolves that were less hostile towards humans would essentially domesticate themselves by naturally selecting for tamer wolves since that would allow for easier access to food from human settlements. A major critique of the latter hypothesis is whether evolution by this natural selective pathway could have occurred in a sufficiently short time span. Simulating the process would help demonstrate if such an objection is sufficient to dismiss this hypothesis. Thus, we constructed an agent-based model of the evolution of a single trait, a measure of human tolerance, in canines to test the merit of the time constraint objection. We tested scenarios both with and without mate preference to provide a potential sexual selective force. We used fecundity and mortality rates from the literature for validation. Hartigan’s dip test of unimodality was used to measure if and when divergence of populations occurred. Our results indicate that the proto-domestication hypothesis cannot be rejected on the basis of time constraints.

## 1. Introduction

There is strong archaeological [1–3], acoustic [4], genetic [5–7] and behavioural [8] evidence that dogs (*Canis familiaris*) are the earliest domesticated animals [9,10] with a predominant, or perhaps unique, ancestor: ancient wolves. There exist two historical periods in which ancestors of grey wolves (*Canis lupus*) were domesticated into dogs [11]. The more recent period, 15 000 BP (calendar years before the present) to the modern era, has strong evidence for artificial selection pressures from humans [2]. The earlier period, approx. 30 000 to 15 000 BP contains archaeological evidence of prehistoric dogs [12], but the method of domestication is not as obvious and has been a topic of debate in the literature [9]. Notably, recent genetic studies have pushed this potential origin date of dogs back to 40 000 BP [6].

On one side of this debate is the theory that both domestication periods resulted from similar artificial selection pressures. One such conjecture is the hunting hypothesis, which states that wolves were domesticated through means like aggression reduction to aid humans in hunting [13]. While there is significant evidence for this hypothesis in the more recent era of domestication, the support for such reasoning weakens in the earlier domestication period. In the earlier domestication period, humans hunted by using direct impact killings, rather than methods that would encourage tracking or retrieval, such as the arrows used in the later domestication period. This reduces the assistance that wolves could provide [11] to the already successful hunting techniques early humans possessed [14]. There is also evidence that prehistoric wolves and humans had overlapping niches leading to competition, as both humans and wolves were family hunters of large herbivores and were prone to territorial defence, which questions the motivation for domestication rather than eradication attempts [14].

Another conjecture based on early artificial selection is the pup-adoption hypothesis, which posits that humans adopted and hand-reared wolf pups, imprinting on them and forming bonds [15]. These wolf pups were socialized with humans, and the pups which failed to socialize were probably culled. As a result, well-socialized and tamer wolves were raised to sexual maturity, with their offspring being raised similarly. These tamer wolves became reproductively isolated from aggressive, non-socialized wolves and successive generations of pup adoption, socialization, feeding and reproductive isolation, led to the formation of dogs [16,17]. This hypothesis suffers from a few objections, such as scarce archaeological evidence of pet-keeping during this time period, an implicit assumption of surplus resources for humans to share with wolf pups, and the evolutionary benefits of humans to rear wolves is unclear [16,18].

The other side of this debate is that wolves domesticated themselves through natural selection pressures. Often summarized using the proto-domestication hypothesis (also known as the commensal scavenger hypothesis or self-selection hypothesis), this theory suggests that a portion of prehistoric wolves began to scavenge food from prehistoric human settlements. This method provided a relatively easy and consistent food source in comparison to fluctuating wild food sources. In doing so, these wolves required less adrenaline, i.e. were less aggressive or apprehensive, which increased their tolerance and preference to living close to early humans. These wolves then colonized the human-occupied environments and, from this group, emerged the first primitive dogs [19,20]. A number of objections exist against this hypothesis: (i) humans did not produce sufficient food scraps to provide a reliable ecological niche for tamer wolves to use [21]; (ii) humans would choose to remove human-tolerant wolves because they could have been considered threats [16]; and (iii) the natural selective pressures are not strong enough for speciation to occur in such a short time period [10]. This proto-domestication hypothesis is the focal point of our research, where we choose to model this dynamic process and investigate the merit of the time constraint objection.

Famous observations by Darwin, studies by Belyaev and colleagues on silver foxes [22] as well as work on neural crest cells [23] (although this neural crest cell hypothesis has recently undergone criticism [24]) point to the ability to select for one characteristic, tameness, to result in a number of outcomes for seemingly unrelated traits (e.g. floppy ears, shorter snouts, etc.) collectively called domestication syndrome [25]. Even if on its own tameness does not result in all aspects of domestication syndrome, we can consider it a principal component of a higher-dimensional, multivariate phenomenon. Because of this, our model will use the mathematically and computationally simplifying assumption of single-trait evolution.

Traditional mathematical models in ecology and evolutionary biology are based on systems of differential or difference equations. In these models, fitness effects and population structure are represented in ways that are simplified and ignore the individual organisms’ natural behaviour in favour of overall population dynamics [26]. Hence, we choose to simulate wolf domestication by natural selection using an agent-based model (ABM). ABMs consider individuals with unique properties rather than population-level, structured models [10]. These individual units interact using explicit rules which then result in emergent properties such as evolution.

Our ABM includes user-defined parameters such as mutation rate, fecundity values, carrying capacity, annual (non-starvation) survival probability and food distribution between wild food sources and human food sources. It outputs the population distribution of a single trait, wolves’ tolerance of humans, over time. The model also includes human development to drive niche competition [27] and cooperative breeding [28]. The ABM contains two potential drivers of evolution: starvation and female mate selection, the latter of which is turned on or off by a user setting. Additionally, we examine two potential scenarios for human settlement in the landscape: when human presence is constant over the time period and when human presence increases linearly. We then analyse under which parameter sets the wolf population diverges into two distinct sub-populations, where one is more human-tolerant than the other. Previous studies exist using agent-based modelling of single-trait evolution (e.g. [29,30]), and of wolves in general [31–33], but to the authors’ knowledge, no ABMs exist simulating the evolution of dogs from wolves via natural selection.

We investigate if, in the use of an ABM constructed of biologically supported rules of behaviour, evidence of the self-domestication of wolves could emerge in such a short period of time. Importantly, our methods neither prove nor disprove the proto-domestication hypothesis, rather, we determine if the time constraint objection is sufficient to dismiss the proto-domestication hypothesis, or if additional objections are necessary.

## 2. Methods

An agent-based model is a computational tool for simulating the actions and interactions of autonomous individuals, called agents, among themselves, as well as their interactions with their environment. Our ABM is constructed using Python 3.10 [34]. The model description follows the overview, design concepts, details (ODD) protocol for describing individual- and agent-based models [35] as updated by [36]. The ODD protocol contains seven subsections (called elements) of increasing detail which are provided below. The Dryad repository containing all scripts is provided in the Data accessibility section below.

### (a) Purpose and patterns

The purpose of this model is to test the feasibility of the proto-domestication hypothesis. The proto-domestication hypothesis, as mentioned earlier, is that a sub-population of prehistoric dogs emerged from wolves during a 15 000 year period through selective forces that were conducive for high degrees of human tolerance. The feasibility of the proto-domestication hypothesis will be supported if the model suggests that prehistoric canines would rapidly develop and sustain two opposing human tolerance strategies, both tolerant (prehistoric dogs) and not tolerant (wolves) through entirely natural selective forces.

The patterns recorded in our model are observations used to deem our model credible to serve this purpose. We measure two necessary patterns, the persistence of canines and their age distribution. Since prehistoric canines gave rise to modern canines, it is necessary that our model predicts that a prehistoric canine population would persist throughout the 15 000 year period and emerge with an age distribution that is similar to those measured empirically.

### (b) Entities, state variables and scales

The main entity in our model is the agents, in our case, canines. There is no explicit spatial dimension of our model, instead, we assume all canines exist in a shared environment and include a single entity for the amount of food units.

Canines have three state variables: age (an integer value between 0 and 10 representing the number of years the canine has survived), sex (male or female), and human tolerance (a value between 0 and 1 measuring the degree of dog-like qualities). Canines with a human tolerance value near zero exhibit traditional wolf-like behaviour, whereas canines with a human tolerance value near one exhibit dog-like behaviour, such as reduced flight distances from humans and reduced aggression.

The shared environment has two state variables: the total number of food units (an integer between zero and a parameter that represents the capacity of the environment) and the proportion of food units that are of human food type (a value between 0 and 1 measuring the proportion of the resources that are only accessible by scavenging from humans). One time step, also referred to as a ‘tick’, represents one year and simulations were run for 15 000 years, or until the number of canines reached zero. The absence of explicit modelling of humans as agents, human settlements as collectives, social rankings and pack dynamics of canines, or spatial relationships was done for simplicity, all of which present plausible extensions of the model.

### (c) Process overview and scheduling

Each year the model executes the following processes in a random (unless otherwise stated) execution order of canines:

1. The environment executes the ‘environment update’ submodel, which updates the total amount of food and the proportion of the total food that is of human type and then partitions the total food into human and wild types.
2. The ‘hunt’ submodel is performed with all canines, which includes:
  - All canines compete to obtain a human food unit, with canines having large human tolerance values being more competitive to obtain those units.
  - All canines that were unable to obtain a human food unit compete to obtain a wild food unit, with canines having small human tolerance values being more competitive to obtain those units.
  - All canines unable to obtain a food unit are removed due to starvation.
3. The ‘non-starvation death’ submodel is performed with all canines, which is a random process to determine if the canine suffers a non-starvation related death.
4. The ‘reproduce’ submodel is performed with all canines, which includes:
  - Identifying which canines are sexually mature based on their age.
  - Each sexually mature female is assigned a probability of reproducing dependent upon the current environmental conditions. The sexually mature females determine if they will attempt to find a mate according to this probability.
  - Those females that are selected to attempt to find a mate are paired with a sexually mature male, which are assigned either randomly or based on the similarity of their human tolerance values.
  - The number of pups in each litter is computed, accounting for pup mortality. Each pup is generated and assigned state variables (age, sex, and human tolerance).
5. If there are less than three canines, the model terminates due to the impending extirpation.
6. If the year is a multiple of 10, a statistical test for speciation in the present year is conducted, with the *p*‐value, amount of food units, and the state variables of all canines recorded into a single census for processing results.
7. Each canine has their age incremented by a single unit.

### (d) Design concepts

#### (i) Basic principles

This model leverages a principle known as domestication syndrome [25] which suggests that selection for a single trait, here, human tolerance, can result in multiple outcomes of other traits (e.g. floppy ears, shorter snouts, etc.). At its core, measuring the feasibility of the proto-domestication hypotheses is determining if plausible selection pressures on human tolerance are sufficiently strong to provide an ecological niche for the development of prehistoric dogs within a short time period.

#### (ii) Emergence

The primary emergent behaviour of interest is the distribution of human tolerances among canines. This distribution varies throughout the temporal simulation of the model and is driven by naturally selective forces, and optionally, sexually selective forces (referred to as ‘mate preference’ in our model). For example, canines that adapt a generalist strategy are relatively more competitive to obtain either food type during their hunting, but less competitive for any particular food unit. Furthermore, when mate preference is enabled, canines with a popular human tolerance strategy have an increased likelihood of mating, which in turn increases the similarity of human tolerance strategies between subsequent generations. The limited lifespan and chance mortality that is not affected by human tolerance can diminish the capacity for novel strategies to persist into a stable breeding sub-population.

Secondary emergent behaviours are the persistence of canines and their age distribution. These results are more imposed than the human tolerance distribution, due to selecting parameters that result in a fairly stable amount of food and using empirical survival probabilities for non-starvation mortality.

#### (iii) Adaptation

Canines consume whichever food unit is first available to them, only attempting a second feeding if they failed to obtain the first food unit (an example of indirect objective seeking). To reflect alloparental care and the high biological cost associated with reproducing in a resource-depleted environment, female canines are more likely to forgo their own mating if the number of canines is relatively close to the capacity of the environment (an example of direct objective seeking). If mate preference is enabled, females may also avoid mating with males with significantly different human tolerance values, and have a weighted probability of mating with the remaining sexually mature males (an example of direct objective seeking).

#### (iv) Objectives

The decision to attempt a mating for a female is a direct objective that attempts to minimize the number of litters necessary to reach the carrying capacity of the environment. We use the logistic difference equation as a basis to calculate the probability of reproducing for each female, solving for the per-litter contribution as a proxy for this probability (see §2g(iii) for more details).

When mate preference is enabled, the mate selection algorithm aims to reduce, on average, the difference in human tolerance values between mates. Females who elect to attempt a mating assign a probability to mating with each male, where males having similar human tolerance values to the female have a higher probability of being chosen (see §2g(iii) for more details).

#### (v) Learning

Learning is not implemented.

#### (vi) Prediction

The only implicit prediction is done when female canines estimate the number of litters the environment can support before choosing to mate. More rationale and details are provided in §2.

#### (vii) Sensing

Canines are assumed to be aware of the ages and sexes of all canines (including themselves) only to assess sexual maturity. All canines are able to sense if and when they have obtained a food unit, which food unit they are attempting to consume, and if there is a non-zero amount of that food type. Further, female canines are assumed to be aware of the total number of canines.

#### (viii) Interaction

Canines directly interact with the environment by consuming food units (at most one per year). Canines directly interact with each other through mating. Mediated interaction occurs when canines compete for a limited number of food units and mates within each year.

#### (ix) Stochasticity

Stochasticity is used in initializing the model (see §2e) to assign initial ages, human tolerances and sexes to canines. Stochasticity is also used when updating the amount of food in the environment to allow for fluctuating resources due to external factors (e.g. weather volatility, diseases in prey, etc.). Further, stochasticity is used to select the order in which canines attempt to consume these limited resources, in addition to the probability they are successful in obtaining these resources. These processes have a random component to allow for canines without optimal human tolerances to still be able to consume resources.

Stochasticity is also included in the reproduction process, including the likelihood females seek mates, the mate selection process and the number of pups generated from each litter. These processes are all modelled as stochastic to reflect the underlying biology, where females may make sub-optimal mating decisions and have irregularly sized litters.

The pups generated from each litter also undergo a stochastic processes with the assignment of their sex. Each pup also has a random chance of suffering natural mortality, reflecting the relatively high degree of mortality of pups that is difficult to observe. Further, each pup has a chance of being deemed a mutant, whereby they can obtain a human tolerance value substantially different from their parents, otherwise, they are randomly assigned a human tolerance value close to their parents. This stochastic process is necessary for the evolution of variability in human tolerance, otherwise, a process that confines a pups’ human tolerance to be between the values of their parents’ will result in a convergence to a single human tolerance uniformly throughout the population.

All canines can suffer an age-dependent non-starvation related mortality, which is implemented through a stochastic process. This process reflects the plausible situation under which a canine would suffer mortality from an event not captured in the hunting submodel, such as disease, intraspecific competition, etc.

#### (x) Collectives

The collective of interest is if a group of human-tolerant canines can naturally emerge from the models' predefined rules and the behaviors of agents. If so, this human-tolerant group could be referred to as prehistoric dogs.

#### (xi) Observation

The main quantity of interest to observe is the distribution of human tolerance values. In particular, we want to assess whether this distribution is unimodal, suggesting that canines are continuing to use a single strategy (as initialized), or if the distribution is multimodal, suggesting that canines are using multiple strategies (not as initialized) and may indicate a speciation event.

Every 10 years (due to computational cost), we perform Hartigan’s dip test of unimodality to assess if the distribution of human tolerance deviates from unimodality in a manner that exceeds random chance (a *p*-value less than 0.05). After the model finishes simulating 15 000 years, we perform a backwards search (starting from final year of simulation) to determine if there exists a speciation event. A speciation event is deemed to have happened if we observe 1500 consecutive years where we reject unimodality and support multimodality. We chose 1500 consecutive years (10% of the time extent of the model) as a conservative estimate for how long behaviour must remain distinct (multimodal) for speciation to affect physical traits enough for a speciation event to be plausible. In the case there is multiple speciation events in a single run, we only record the latest such event. Each speciation event also has the duration of the event recorded (in number of years) and the year when the first multimodality was statistically significant.

### (e) Initialization

The model is initialized by setting the amount of food units and number of canines equal to the environmental capacity ($F_{0}=C_{0}=c$). Each canine is assigned a random sex (with equal probability). Each canine is assigned an age using the age distribution found in [37] assuming a low hunting pressure, using a probability mass function given in the electronic supplementary material

The human tolerance values for each canine ($\tau_{i}$) is assumed to follow a truncated normal distribution on the unit interval, with mean $\mu_{\tau }$ and s.d. $\sigma_{\tau }$,


(2.1) $$\tau_{i}\overset{iid}{∼}N_{\left[0,1\right]}\left(t_{i};\mu_{\tau },\sigma_{\tau }^{2}\right),\;\text{ for }i\in \{1,\ldots ,c\}.$$

### (f) Input data

The user must specify two modelling forms for how (i) the presence of humans alters the proportion of resources that can be scavenged by canines throughout time, referred to as the ‘human food style’, and (ii) if the likelihood of mate selection is a function of similar human tolerances, or completely random, referred to as the ‘mate selection’ process.

We allow the user to choose between two human food styles, adopting either the assumption that (i) the proportion of food that could be scavenged from humans remains roughly constant, or (ii) it increases linearly.

We allow the user to choose between two mate preference styles, either (i) all sexually mature males are equally likely to be chosen for a reproductive female, or (ii) only males who have similar human tolerance values are deemed ‘competitive’ (i.e. a higher probability of being selected) for mating.

### (g) Submodels

#### (i) Update environment

The total amount of food in the environment at time t, denoted $F_{t}$, is drawn from a truncated normal distribution (from 0 to the capacity of the environment, c) centered around the previous years’ amount of food with standard deviation $\sigma_{F}$,


(2.2) $$\begin{array}{lcr} & F_{t}∼\left[N_{\left[0,c\right]}\left(F_{t-1},\sigma_{F}^{2}\right)\right],\;\;\text{ for }t\geq 1, & \end{array}$$

where [⋅] is the nearest integer function.

The amount of human type food is computed as $\left[h_{t}F_{t}\right]$, where $h_{t}$ is the proportion of the total food that is human type, a function specified by the user. The user has two options for the function $h_{t}$, either a constant function (suggesting the resource partition between human and wild-type food did not change over this time period), or a linear increasing function (suggesting a constant increase in the proportion of resources provided by humans). For comparability, we ensured the accrued proportion of resources that are of human type is equal (the area under the curve of both $h_{t}$ functions is the same), using, $h_{t}=h_{\max}/2$ for all t in the constant setting and $h_{t}=\frac{t}{15\;000}h_{\text{max}}$ in the linear increasing case. The amount of wild-type food is computed as $F_{t}-\left[h_{t}F_{t}\right]$.

#### (ii) Hunt

The hunting action is divided into two parts. In the first part, all canines will hunt for human-type food, with those canines who do not attempt a feeding and those who fail their feeding being permitted to enter a second round hunting for wild-type food.

During the hunt for human-type food, canines are selected to attempt a feeding based on a weighted-random process that favours more human-tolerant canines to have earlier attempts. In particular, the model deterministically sorts canines in decreasing order of human tolerance (τ) values. Let X denote this set of canines who are still pursuing a food unit. While the number of human food units remains positive, the following actions are taken:

1. A uniform random number is generated between 0 and the sum of the human tolerance values of all remaining, non-fed, canines $u∼U\left[0,\sum_{i\in X}\tau_{i}\right]$.
2. Each canine corresponds to a segment of the number line sample from part (1) corresponding to their human tolerance. Thus, the random number drawn in part (1) identifies a canine by the segment it belongs to, that is, the smallest value of j∈{1,…,card(X)} such that, $\sum_{i=1}^{j}\tau_{i}\geq u$. Thus, a particular canine is identified and attempts a feeding.
3. The canine attempting a feeding has success with probability $\tau_{i}$, that is, the higher degree of human tolerance, the more likely they are to be successful in obtaining their human food unit.
4. The canine is removed from the set X. If the canine was successful, they survive this hunt process and will not perform a wild-type hunt. If the canine was not successful, they will hunt wild-type food.

The human food hunt concludes in either two situations: (i) all canines are fed, or (ii) some canines are not fed (meaning they were either not selected before human food units were depleted, or, they were unsuccessful when granted the opportunity to hunt human type food). The canines who are not fed are allowed to hunt wild-type food, where the process is identical to the human type food except for the replacement of $1-\tau_{i}$ instead of $\tau_{i}$ (providing an advantage for non-tolerant canines to feed). Any canines not fed after the wild food hunt are assumed to have starved and are removed from the model.

#### (iii) Reproduction

We assume the probability a female reproduces depends on the relative abundance of canines to the resource capacity of the environment. We assume the number of canines after breeding, $C_{t+1}$, can be computed as a function of the number of canines before breeding, $C_{t}$, using the logistic difference equation,


(2.3) $$\begin{array}{lcr} & C_{t+1}=C_{t}+rC_{t}\left(1-\frac{C_{t}}{c}\right), & \end{array}$$

as motivated by the cooperative breeding styles of canines [28]. Thus, when the number of canines, $C_{t}$, approaches the capacity of the environment, c, the number of new canines approaches zero, $C_{t+1}-C_{t}\approx 0$, reflecting alloparental care and mitigation of risk of reproducing in a resource-depleted environment. We can solve for the per-capita growth,


(2.4) $$\begin{array}{lcr} & \frac{C_{t+1}}{C_{t}}=1+r\left(1-\frac{C_{t}}{c}\right). & \end{array}$$

The fraction of surviving litters can be found by dividing the per-capita growth contribution by the average number of pups in a litter, $\mu_{L}$, and the probability that a pup survives to independence, q, to be,


(2.5) $$\begin{array}{lcr} & \frac{1+\left(1-r\frac{C_{t}}{c}\right)}{q\mu_{L}}. & \end{array}$$

Since we assume males do not have a probability of attempting to reproduce, we limit the above expression to female canines, replacing $C_{t}\mapsto C_{t}^{♀}$ and the contribution of females to the environment $c\mapsto c_{t}^{♀}$, finding the probability a female reproduces as,


(2.6) $$\begin{array}{lcr} & L_{t}=\frac{1+\left(1-r\frac{C_{t}^{♀}}{c_{t}^{♀}}\right)}{q\mu_{L}},\;\;c_{t}^{♀}=\frac{C_{t}^{♀}}{C_{t}}c. & \end{array}$$

Thus, the submodel begins with all sexually mature (at least two years old) females [14,38] assigned a probability of reproducing, $L_{t}$. Canines never reach reproductive senescence [14]. To assign a mate, a user-specified choice of either ‘no mate preference’, or ‘mate preference’ is used to determine if there is a sexual selection pressure for similar human tolerances. In either case, mates are assigned sequentially to sexually mature females in a random order, halting when no sexually mature males, those that are least 2 years old, are available or all females have found a mate. Note: we assume all mating pairs are annually-monogamous [39] which are not consistent between years to (i) avoid pair tracking, and (ii) indirectly include the effects of casanova canines [40], re-selection of dominance hierarchies [41] and possible plurality of fathers within a single litter [41].

If ‘no mate preference’ is enabled, each sexually mature male has an equal chance of being selected for reproducing with each sexually mature female. If ‘mate preference’ is enabled, the female has a pickiness, p, which defines the set of possible males for her to reproduce with, that is,


(2.7) $$\begin{array}{lcr} & M_{♀}=\{♂:|\tau_{♀}-\tau_{♂}|<p\}. & \end{array}$$

Each male in the set $M_{♀}$ is then assigned a probability of being selected based on the similarity of their human tolerances relative to all males in the set $M_{♀}$,


(2.8) $$\begin{array}{lcr} & \frac{1-|\tau_{♀}-\tau_{♂}|}{\sum_{i\in M_{♀}}1-|\tau_{♀}-\tau_{♂,i}|}. & \end{array}$$

The chosen male and female reproduce and are removed from their potential mating lists.

The mating pair produces a litter of size [S] drawn from a normal distribution, $S∼N(\mu_{L},\sigma_{L}^{2})$. Each pup is assigned an age of zero, a random sex and a human tolerance value. The assignment of human tolerance is typically drawn from a uniform distribution between the sire and dam’s human tolerance values with a small amount of environmental noise $\sigma_{e}$,


(2.9) $$\tau ∼U[\min(\tau_{\text{♂}},\tau_{\text{♀}}),\max(\tau_{\text{♂}},\tau_{\text{♀}})]+N(0,\sigma_{e}^{2}),$$

which is constrained between 0 and 1 in the unlikely event that the noise pushes the tolerance outside the feasible range. However, with probability m, a pup is deemed to be a ‘mutant’, and instead of being limited to a tolerance between their parents, has an equal chance of being assigned any possible human tolerance, τ∼U[0,1].

Due to relatively high pup mortality [42] each pup is assigned a probability of surviving beyond this stage q. Pups that do not survive are immediately removed.

#### (iv) Non-starvation death

Not all mortality is due to starvation. Canines suffer natural mortality through other sources of intraspecific competition (e.g. aggression during mating), interspecific competition, disease and other factors that are not fully accounted for or entirely missing from the mortality in the hunting submodel. Therefore, we estimated the percentage of mortality events that arise from non-starvation-related causes (see electronic supplementary material for details and [43]) and the age-dependent probability a canine suffers mortality (see electronic supplementary material for details and [44]). Therefore, we can find the joint probability of a natural death occurring for a canine dependent on age,


(2.10) P(natural death at age a)=P(death at age a)P(natural death|death at age a)=L(a)⋅0.67,

where the L(a) function is estimated in the electronic supplementary material.

(v) Age update

The final submodel simply increases the ages of all canines by 1 year.

## 3. Results

### (a) Model verification and validation

To verify our model’s capabilities, we demonstrated our model was capable of producing a robust population of prehistoric dogs under the extreme parameter configuration, $h_{15000}=1$, that is, human food completely saturates the food supply by the end of the 15 000 year time period. Next, we verified that with equal food proportions, $h_{t}=0.5$ for the entire 15 000 year time period, mate preference selection forces a separation of human tolerance values that is sustained throughout the simulation. Finally, we verified that with equal food proportions, $h_{t}=0.5$ for the entire 15 000 year time period, the absence of mate preference fails to give rise to any sustained adoption of extreme human tolerance strategies. These three verification techniques ensured our model is capable of producing reasonable dynamics that could either support or oppose the proto-domestication hypothesis. We also validated that the model predicted a similar age distribution to that found in present day wolves [45] for all combinations of mate preference and human food forms. Further details, figures and discussion are contained in the electronic supplementary material for reference.

### (b) Default parameter values

**Figure 1.**
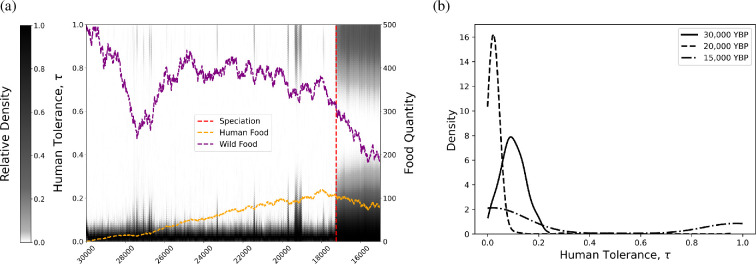
(a) Density of human tolerance values for a single model repetition at default parameter values. Tolerances with a higher density indicate a larger number of canines with that trait value. The red line indicates the time of speciation defined by at least 1500 consecutive years of statistically significant multimodality according to Hartigan's dip test. The yellow and purple lines display the random walk of the amounts of human and wild food, respectively. (b) Distribution of human tolerance values for the same model repetition at various time points (line style). The default parameter values, mate preference and increasing human food model choices were made to generate the time series.

Using default parameter values, speciation was found in 37.1% of all repetitions. With mate preference turned on in the model, we found speciation in 74.2% of repetitions (66.2% with constant human food and 82.2% with increasing human food) compared with 0% of repetitions without mate preference. The output from an example repetition at default parameter values can be found in figure 1. These results suggest that our speciation algorithm is able to detect and properly time a speciation event as intended, that is, the time at which two sustained sub-populations of differing human tolerances are formed. The distinct sub-populations not tolerating or highly tolerating humans can be clearly seen in bimodal distribution in figure 1*b*. Furthermore, we note through various simulations of the model, speciation events tend to occur near times of relatively limited resources, such as in figure 1*a*, suggesting a selective pressure to adapt a strategy that promotes a greater access of resources.

**Figure 2.**
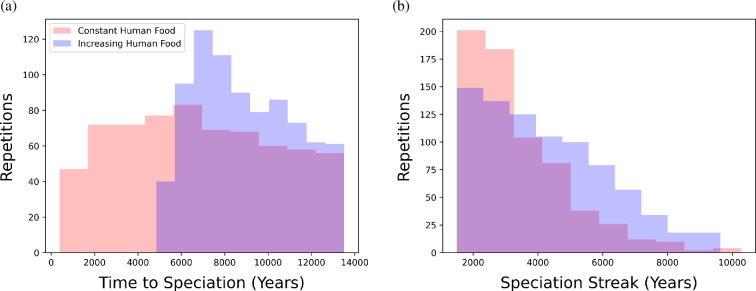
(a) Distribution of the number of years simulated at default parameters until speciation occurred. (b) Distribution of the number of years for which distinct sub-populations were retained at default parameter values, that is, the speciation streak. A total of 1000 repetitions were simulated for each human food style (colour), with up to 50 realizations per repetition until persistence was recorded. All repetitions displayed were done at default parameter values, however, only repetitions with speciation are included in these figures. Note that due to no speciation in the absence of mate preference, only repetitions with mate preference are shown.

With mate preference, the median time to speciation was 8030 years (6850 years with constant human food and 8976 years with increasing human food). Furthermore, with mate preference the median duration of speciation was 3425 years (2990 years with constant human food and 3945 years with increasing human food). The distributions of time to speciation and the number of years for which distinct sub-populations were retained (the ‘speciation streak’) are provided in figure 2.

### (c) Sensitivity analysis

To select a proper sensitivity analysis methodology for each of our speciation metrics (speciation percentage, time to speciation and speciation streak), we assessed the monotonicity of each metric with respect to changes in each parameter within ranges according to table 1, detailed in the electronic supplementary material. We found that speciation percentage was non-monotonic with respect to changes in parameter values, whereas time to speciation and speciation streak were approximately monotonic. Therefore, we elected to use the extended Fourier amplitude sensitivity test (eFAST) as an index for sensitivity of speciation percentage and partial rank correlation coefficient (PRCC) as an index for sensitivity of time to speciation and speciation streak. We also found that speciation never occurred in the absence of mate preference, therefore, all further simulations were conducted with mate preference, suggesting it may be a necessary component for the proto-domestication hypothesis.

**Table 1.** Parameters and states are fixed in time unless indicating time dependence by a subscript of t. † indicates a parameter varied logarithmically when sampled.

parameter/state	description	default Value	range	source
τ	a canine’s tolerance of humans (agent variable)	$N(\mu_{\tau },\sigma_{\tau })[0,1]$	N.A.	
$\mu_{\tau }$	mean of initial human tolerance	0.1	not varied	
$\sigma_{\tau }$	s.d. of initial human tolerance	0.2	not varied	
$\mu_{L}$	mean number of pups per litter	6.8	20%	[46]
$\sigma_{L}$	s.d. of number of pups per litter	2.2	20%	[46]
r	growth rate	4.08	20%	[46]
q	probability a pup survives first year	0.5	0.4 - 0.6	[42]
m	probability of mutation in tameness	$10^{-2}$	$10^{-3}-10^{-1}†$	
$\sigma_{F}$	s.d. of total food random walk	$10^{0.3}$	$10^{-2}-10^{0.5}†$	
$h_{\text{max}}$	maximum human food proportion	0.3	0.01−0.5	
c	total food carrying capacity	500	400−600	
p	mate pickiness	0.2	0−0.4	
d	prob. a death is due to non-starvation causes	0.68	20%	[43]
$C_{t}$	number of canines	$C_{0}=c$	N.A.	
$F_{t}$	total amount of food	$F_{0}=c$	N.A.	
$h_{t}$	proportion of total food of human type	varied	constant/increasing	
$L_{t}$	probability a female reproduces	calculated	N.A.	

**Figure 3.**
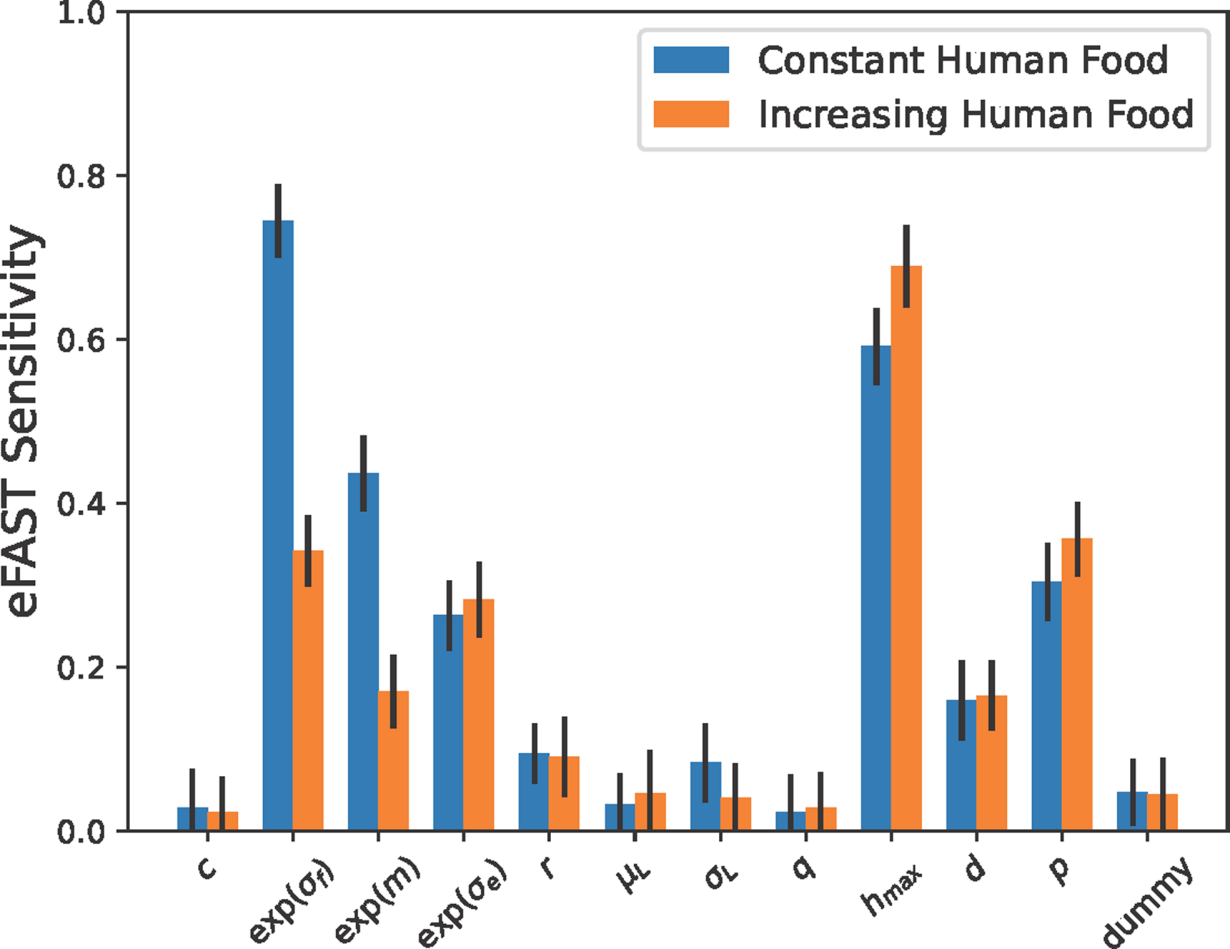
eFAST total-order sensitivity indexes for each parameter varied in table 1 for the percentage of repetitions that result in speciation. For each human food style (colour), we used a number of parameter sets, $N=N_{S}\times k\times N_{R}=(65\times 12)\times 12\times 1$, with 20 repetitions per set, leading to 374 400 model realizations. Repetitions were repeated up to 50 times until persistence was recorded. We used an inference parameter of four for the Fourier series decomposition. Error bars denote 95% confidence intervals. Note that due to no speciation without mate preference, this figure is generated from repetitions that assume mate preference.

We found whether speciation occurred or not to be most sensitive to the standard deviation of the bounded random walk for resources ($\sigma_{f}$), the proportion of food that is human type ($h_{\text{max}}$), the probability of mutations (m), the degree of ‘pickiness’ displayed by females to choose a mate with a similar human tolerance (p) and the s.d. of the environmental perturbation to a non-mutated pup’s tolerance ($\sigma_{e}$). The relative sensitivity of the model to these parameters was similar for each human food type with two major exceptions: the volatility in the resources and the percent of mutations was significantly more important under a constant human food assumption. Each empirically estimated parameter (c, r, $\mu_{L}$, $\sigma_{L}$, q, d) was relatively unimportant on whether or not the model speciated when compared to a ‘dummy’ parameter that was unused in simulations (see figure 3). Across all repetitions in the eFAST analysis we found a speciation percentage of 6.7% (3.5% with constant human food and 10.0% with increasing human food), suggesting our wide ranges of parameter values in table 1 contain parameter regimes where speciation is rare, in particular, these are likely to occur in regimes where resources are reliable, mutations are rare and human food makes up a small proportion of resources.

**Figure 4.**
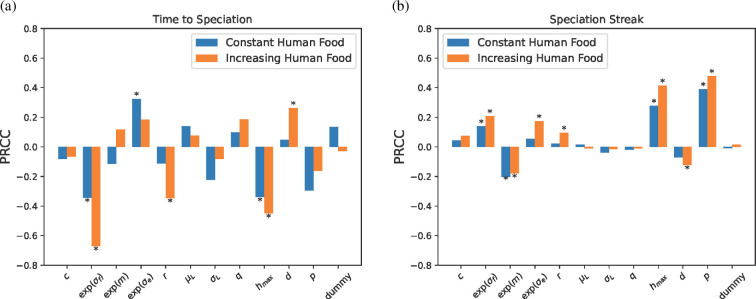
(a) Partial rank correlation coefficients (PRCC) for time to speciation averaged across 20 repetitions. (b) PRCC values for the number of years for which distinct sub-populations were retained, called the speciation streak averaged across 20 repetitions. PRCC values marked with an asterisk are statistically different than zero. For each human food style (color) we used N=500 parameter sets with 20 repetitions (which repeated up to 50 times until persistence was recorded), leading to 20 000 model realizations. Note that due to no speciation in the absence of mate preference, these figure is generated from repetitions that assume mate preference.

We found the more volatile environments ($\sigma_{f}$) and higher human food proportions ($h_{\text{max}}$) lead to more rapid and robust speciation events (refer figure 4). Further, increased pickiness during mate selection with respect to similar human tolerance values (p) lead to a longer duration of speciation. Some empirically estimated parameters modestly influenced the time to speciation and speciation streak (r, d) under the assumption of increasing human food, however, these are well-estimated and have relatively smaller PRCC values. Across all repetitions, we found the median time to speciation was 10 825 years (7460 years with constant human food and 11 315 years with increasing human food) with the speciation lasting a median of 3185 years (3385 years with constant human food and 3170 years with increasing human food).

## 4. Discussion

We constructed an ABM of single-trait evolution of canine (i.e. ancient wolves and early dogs) human tolerance over a 15 000-year period in an attempt to determine the validity of the proto-domestication hypothesis. Our model suggests that the proto-domestication hypothesis should not be entirely dismissed on the basis of the corresponding relatively short time period. A number of plausible objections to the proto-domestication hypothesis remain, including questions with respect to the amount of human food scraps produced and the tolerance of humans to human-tolerant wolves.

We validated the dynamics of our model were similar to modern wolf dynamics, scrutinizing the age distribution of canines as an emergent property. The distribution of ages of canines was shown to not differ significantly from an empirical distribution from the literature [45]. Thus, the birth and death dynamics in our model are realistic.

At default parameter values, assuming the model form of mate preference and an increasing human food percentage, canines quickly develop a preference for the relatively abundant wild food. As the human food percentage slowly increases, a small number of canines begin to adopt the opposite strategy (i.e. dogs), with near complete preference for human food (τ≈1) around 19 500 years before present. By 17 200 years before present, the Hartigan’s dip test rejects unimodality consistently for a 150 year time period, suggesting two distinct sub-populations. These sub-populations persist throughout the remainder of the simulation, growing in numbers around 16 800 years before present. See figure 1*a*,*b*.

We employed two different human food styles (constant versus increasing) in our model because it was unknown which was a more accurate model of ancient human effects on the ecology of ancient wolves. The effect of human food style on speciation in our model was that increasing human food over time led to more speciation on average, however it delayed the onset of the speciation. The duration under which two distinct sub-populations of canines coexisted was slightly shorter for increasing human food because of the later onset of speciation. These results are expected. The increasing human food model form initializes the simulation with very little room for an alternative niche that grows over time to be a doubly large secondary niche compared with the constant human food model form. Ultimately, both human food styles allowed for speciation, and in relatively similar amounts, so the uncertainty of which model form is more accurate is of little importance.

A number of simplifying assumptions were employed because of lack of data or to avoid excessive computational complexity. For example, explicit climate dynamics could have been included in the model instead of using the random walk of available food as a proxy. Foremost among our simplifying assumptions was the lack of a spatial component to the model. Including heterogeneity among the human-provided resource landscape as well as in the distribution of the canine agents themselves would provide unpredictably different speciation results. With a spatial component, speciation could be more likely since geographic isolation is a well-known evolutionary driver. However, mutations towards tameness would be less likely to take hold among the population in locations of low human food density, so a spatial component could make speciation more difficult to occur. A future study expanding our model to include such a spatial component would be of interest. Another future direction could be exploring the effects of competition based on sex and size relationships among individuals. Even further, a future study could explore the effects of tameness either enhancing or diminishing survival probabilities. Finally, another expansion of the model could look at ontogenetic effects where an individual could adjust its tameness prior to adulthood.

Our model has two evolutionary drivers: ‘natural selection’ through the two separate ecological niches of wild food and human food, and ‘sexual selection’ when the mate preference routine was employed. A striking result from our study was the necessity that the mate preference routine be employed to witness divergence of the canine populations according to human tolerance values. No simulation runs without the mate preference routine resulted in divergence within a 15 000-year window. Yet, with mate preference, speciation was frequently present. The separate ecological niches alone were simply not sufficient to cause speciation in this time period. Note that ‘sexual selection’ by itself is also insufficient. Thus, it is the combination of both of these evoluationary drivers that allow the canines to speciate within the allotted time frame. This means our model allows the proto-domestication hypothesis to overcome the time constraint objection only if ancient wolves more often selected to mate with wolves with a similar tolerance (or lack of tolerance) for humans. If this preference existed among the ancient wolf population, it implies that human tolerance could be a ‘magic trait’ in that it contributed both to ecological selection and to non-random mating [47,48].

Furthermore, our results suggest that more recent objections to the proto-domestication hypothesis, such that humans failed to produce a sufficient amount of food scraps necessary for this process, may have substantial merit [21]. In particular, our sensitivity analysis suggests that the volume of human food is one of the most important parameters in dictating the amount, speed and robustness of speciation. Therefore, while our work focussed on the time constraint objection to the proto-domestication hypothesis, it also sheds light on the merit of these more recent objections. Such additional objections could be rigorously tested using a potential future extension of our model.

Holistically, the conclusion that the proto-domestication hypothesis cannot be rejected on the basis of a short time period under the necessary condition of mate preference is moderately robust. This is evident due to the non-trivial number of model repetitions that consistently suggested speciation was plausible across a vast range of parameter values. Furthermore, our definition of divergence is conservative, as we require 1500 consecutive years of significant results from the Hartigan’s dip test. If the test had power deviating much from 1, then the effects of type 2 error would compound, substantially reducing the ability to detect divergence.

Our results cannot prove nor disprove the cause of early wolf domestication. Yet, assuming mate preference, 74.2% of repetitions resulted in speciation at default parameters while 6.7% of repetitions resulted in speciation across all parameter combinations during the sensitivity analysis. This quantitatively confirms the proto-domestication hypothesis cannot be rejected due to time constraints alone.

## Data Availability

All code and data are deposited with Dryad and are available at [49]. Supplementary material is available online [50].
